# Glycan detecting tools developed from the *Clostridium botulinum* whole hemagglutinin complex

**DOI:** 10.1038/s41598-021-01501-1

**Published:** 2021-11-09

**Authors:** Ea Kristine Clarisse Tulin, Chiaki Nakazawa, Tomomi Nakamura, Shion Saito, Naoki Ohzono, Keiko Hiemori, Shin-ichi Nakakita, Hiroaki Tateno, Takashi Tonozuka, Atsushi Nishikawa

**Affiliations:** 1 United Graduate School of Agricultural Science, Tokyo University of Agriculture and Technology, Tokyo, 183-8509 Japan; 2 Department of Applied Biological Chemistry, Graduate School of Agriculture, Tokyo University of Agriculture and Technology, Tokyo, 183-8509 Japan; 3 Cellular and Molecular Biotechnology Research Institute, National Institute of Advanced Industrial Science and Technology, Tsukuba, 305-8566 Japan; 4 Division of Functional Glycomics, Kagawa University, Kagawa, 760-0016 Japan

**Keywords:** Glycobiology, Imaging

## Abstract

Lectins are proteins with the ability to recognize and bind to specific glycan structures. These molecules play important roles in many biological systems and are actively being studied because of their ability to detect glycan biomarkers for many diseases. Hemagglutinin (HA) proteins from *Clostridium botulinum* type C neurotoxin complex; HA1, HA2, and HA3 are lectins that aid in the internalization of the toxin complex by binding to glycoproteins on the cell surface. HA1 mutants have been previously reported, namely HA1 W176A/D271F and HA1 N278A/Q279A which are specific to galactose (Gal)/*N*-acetylgalactosamine (GalNAc) and *N*-acetylneuraminic acid (Neu5Ac) sugars, respectively. In this study, we utilized HA1 mutants and expressed them in complex with HA2 WT and HA3 WT to produce glycan detecting tools with high binding affinity. Particularly, two types were made: Gg and Rn. Gg is an Alexa 488 conjugated lectin complex specific to Gal and GalNAc, while Rn is an Alexa 594 conjugated lectin complex specific to Neu5Ac. The specificities of these lectins were identified using a glycan microarray followed by competitive sugar inhibition experiments on cells. In addition, we confirmed that Gg and Rn staining is clearly different depending on cell type, and the staining pattern of these lectins reflects the glycans present on the cell surface as shown in enzyme treatment experiments. The availability of Gg and Rn provide us with new promising tools to study Gal, GalNAc, and Neu5Ac terminal epitopes which can aid in understanding the functional role of glycans in physiological and pathological events.

## Introduction

The sugar structure of glycoconjugates present on the cell surface of different cells change depending on species, tissue, type of cell, cell differentiation or proliferation of disease. One of the challenges in studying glycobiology is how to perform quantitative and qualitative analyses on glycosylation changes in development and disease. Lectins are proteins which can recognize and bind to specific glycan chains and is thus used to complement mass spectrometry analyses of glycosylation profiles. These molecules have been broadly used to identify glycan biomarkers for cancer cells, isolating pluripotent cells, anticancer drugs, antimicrobial agents, and glycan profiling^1–5^. Many lectins, however, have low affinity which leads to technical difficulties in studying glycan structures and in some cases are not useful as analytical tools. With this in consideration, the development of lectins that are stable in solution and possess strong binding properties are of primary importance and are actively being studied.

Multimerization is an approach to increase lectin avidity to targeted glycans. When several lectins are bound together, the binding avidity increases resulting in the detection of low concentrations of glycoproteins several times higher than monomeric lectins^6^. There are several ways to design multimeric lectins, furthermore, there are also lectins that naturally exist as complexes containing more than one sugar binding site, examples of which are the hemagglutinin proteins from the *Clostridium botulinum* neurotoxin complex.

*Clostridium botulinum* is a gram-negative anaerobic bacterium that produces a neurotoxin. Botulinum bacteria are classified into serotypes A to G depending on the antigenicity of the toxin produced. The type C 16S progenitor toxin is composed of a neurotoxin, non-toxic non-hemagglutinin component and several hemagglutinin proteins (HA) designated as HA1 (HA-33), HA2 (HA-17), HA3a (HA-22-23) and HA3b (HA-53)^7,8^. The HA1 and HA3b components are known as adhesins which bind to the mucin-like structures in the microvilli of upper small intestines via sialo- and asialo-oligosaccharides^9,10^. In addition, the HA components play an important role in the uptake and transcytosis of the C16S toxin into an intestinal adenocarcinoma cell line (HT-29) by binding to mucin-like glycoproteins^11,12^. The binding avidities of the HAs contribute to the internalization of the toxin complex into epithelial cells, thereby enhancing the toxicity of NTX.

The sugar binding sites and proteins structures of individual HAs and HAs in complex have already been elucidated in previous reports. Type C HA1 has two binding sites: site I and site II. Site I binds to *N*-acetylneuraminic acid (Neu5Ac), *N*-acetylgalactosamine (GalNAc) and galactose (Gal) while site II binds to galactose only^13^. In terms of HA3, the crystal structure has also been clarified and it is known that HA3b binds to Neu5Ac, especially α2-3, and α2-6-sialyl lactose sugars^14,15^. Furthermore, in the case of the botulinum toxin type C complex, structures of the HA1-2-3 complex in solution have already been elucidated which showed that the complex can be easily reconstructed by mixing each component even in the absence of the neurotoxin protein. We deduced the structure of the complex based on previous reports of crystal structures of BoNT/C HA3^15,16^, BoNT/C HA2-HA3 complex^17^, BoNT/B HA1-HA2-HA3 complex^18^ and whole progenitor toxin^19^.

Previously, we produced HA1 mutants that bind to specific sugars; HA1 W176A/D271F (HA1 WADF) which is specific to Gal and GalNAc and HA1 N278A/Q279A (HA1 NQAA) which is specific to Neu5Ac^13,20^. To create multivalent lectins with increased binding affinity that are specific to select sugars, we conjugated the above-mentioned HA1 mutants with HA2 WT and HA3 WT to create glycan detecting tools from the *Clostridium botulinum* whole hemagglutinin complex.

In this study, two new glycan detecting tools designed to have different binding specificities were produced: namely Gg and Rn. Gg is an Alexa 488 labeled complex with HA1 WADF -HA2 WT-HA3 WT, it has a green fluorophore and binds to galactose, thus we called it Gg. Rn is an Alexa 594 labeled complex with HA1 NQAA-HA2 WT-HA3 WT, it has a red fluorophore and binds to Neu5Ac, thus we called it Rn. This paper (1) describes the protocol used to construct the modified whole hemagglutinin complexes from *Clostridium botulinum*, (2) identifies glycan structures that are specific to these lectin complexes, and (3) shows how these new tools can be used to distinguish glycans present on the surface of different cells.

## Results

### Preparation of new HA complexes

**Figure 1:**
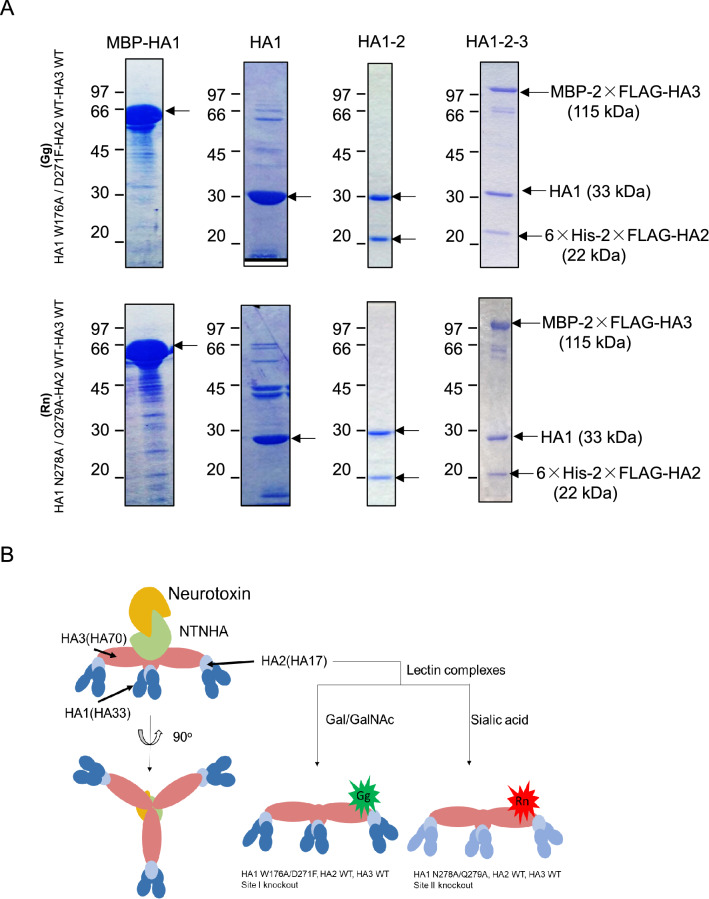
Preparation of HA1-2-3 lectin complexes. (**A**) Proteins obtained at each step of purification were developed on SDS-PAGE. MBP-HA1 lanes shows purified MBP-2xFLAG-HA1 (W176A/D271F (upper), N278A/Q279A (bottom)) used for complex preparation, target protein is indicated by arrows. HA1 lane shows HA1 after MBP tag cleavage (arrow). HA1-2 lane shows HA1-2 complex purified using Ni–NTA affinity chromatography. HA1-2-3 lane shows HA1-2-3 complex after final purification steps. Arrows indicate the bands of proteins MBP-2XFLAG-HA3, HA1, and FLAG-tagged HA2. (**B**) Schematic diagram showing the two lectin complexes produced.

The schematic diagram of the detailed protocol to construct the lectin complex is found in Supplementary Fig. S1. MBP-tagged HA1, 6xHis-tagged HA2 and MBP-tagged HA3 were used for ease in purification, and expression and purification were done separately and consecutively (Fig. 1A). The purified HA1 protein, having a molecular weight of 33 kDa, was collected and dialyzed in preparation for conjugation with HA2. MBP tag was cleaved using enterokinase before conjugation with HA2. Purified HA1 proteins were mixed with HA2 in bacterial lysate because of the inherent low-expression of HA2, by adapting this approach a higher concentration of HA1-2 complex was produced. The resulting HA1-2 complex was confirmed using SDS-PAGE. HA1 having a molecular weight of 33 kDa and 6xHis-T7-2xFLAG HA2 with a molecular weight of about 22 kDa.

MBP-2XFLAG-HA3 protein was expressed separately in *E. coli* and purified using amylose resin affinity chromatography then conjugated with HA1-2 complex. The HA1-2-3 complex was then purified using a Ni–NTA column followed by subsequent purification with an amylose resin column. Bands were confirmed using SDS-PAGE; MBP-2XFLAG-HA3 (115 kDa), HA1 (33 kDa) and 6xHis-T7-2XFLAG-HA2 (22 kDa). Two HA1-2-3 complexes were made, HA1 WADF -HA2 WT- HA3 WT complex and HA1 NQAA-HA2 WT -HA3 WT complex (Fig. 1A, Supplementary Fig. S2).

Finally, the two HA1-2-3 complexes were conjugated with fluorescent dyes. HA1 WADF -HA2 WT- HA3 WT, a galactose and N-acetylgalactosamine selective complex, labeled with Alexa 488 is named as Gg while HA1 NQAA -HA2 WT- HA3 WT, an N-acetylneuraminic acid selective lectin complex, labeled with Alexa 594 is named as Rn (Fig. 1B).

### Glycan affinity of Gg and Rn

**Figure 2:**
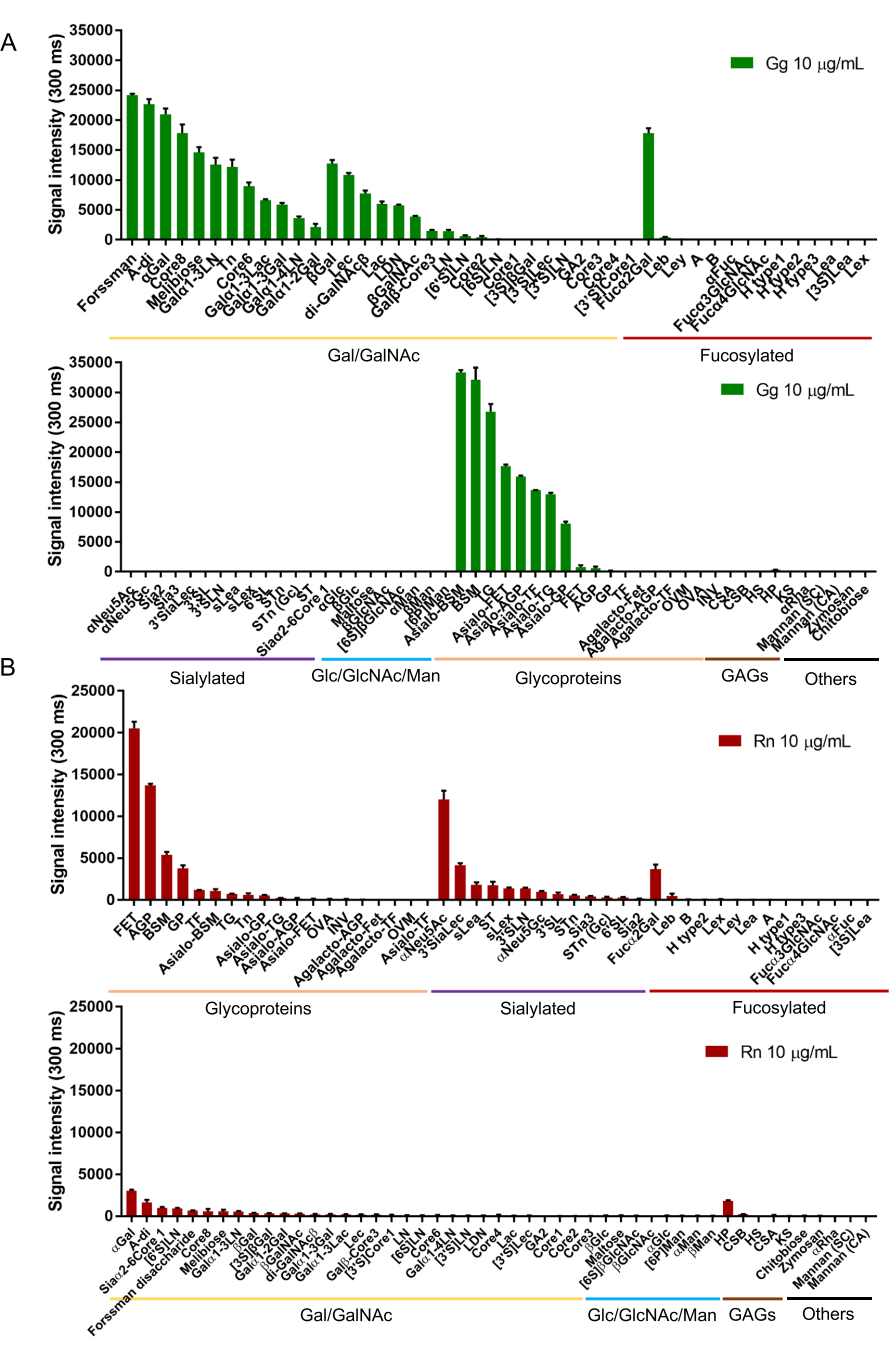
Specificity profiling of Gg and Rn. (**A,B**) Cy3-labeled Gg (**A**) or Rn (**B**) was applied on the glycoconjugate microarray and binding was detected by scanner and analyzed by Array Pro analyzer ver. 4.5. The net intensity value for each spot was determined as the signal intensity minus the background value. Data represents the average ± S.D. of triplicate determinations.

To identify the binding specificities of these two lectins, glycan microarray with an evanescent-field fluorescence assisted detection principle was performed. In concept, 100 glycan structures (Supplementary Fig. S4) were immobilized on a plate and incubated with a Cy3 labeled lectin complex (Gg or Rn). The signal intensity represents the binding strength of the lectin to specific glycan structures. Results showed that Gg has a high binding signal to galactosylated sugars and asialylated glycoproteins (Fig. 2A). Asialo-glycoproteins were prepared by incubating in 0.1 N HCl at 80◦C for 1 h, while agalactosylated glycoproteins were prepared by *Streptococcus* 6646 K β-galactosidase (Seikagaku) treatment. Other terminal sugars such as sialic acid, GlcNAc, Glc, Man, GAGs, sialic acid terminal glycoproteins, and agalactosylated glycoproteins were not labeled. In contrast, Rn has a high binding signal to sialic acid terminal glycans and sialylated glycoproteins (Fig. 2B). Other terminal sugars have very weak to no binding. In addition, both Gg and Rn did not bind to glycosaminoglycans (GAGs) and other more complex polysaccharides like zymosan and mannan. These results highlight the precise specificity of our lectins; Gg binds to Gal/GalNAc terminal glycans while Rn binds to Sialic acid terminal glycans. Images of the glycan microarray plate and plot of binding are shown in supplementary Fig. S3. To further investigate the binding specificity of our lectins, structural models of HA1 and HA1 mutants bound to various ligands were used.

**Figure 3:**
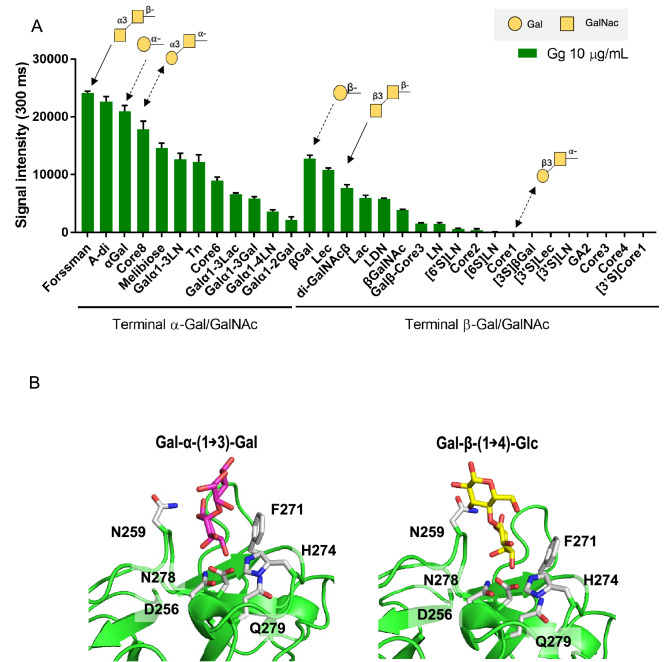
Analysis of Gg specificity. (**A**) Signal intensities of Cy3-labeled Gg to galactose terminal glycans. Glycans with similar structures which only differ in Gal/GalNAc linkage are represented using similar arrows. Cartoon representations of glycan structures are shown on top of each corresponding arrow. (**B**) HA1-D270F model bound to Gal-α-1,3-Gal and Gal-β-1,4-Glc ligands.

To further interpret the results of the glycoconjugate microarray, we took results from Gg analysis and compared binding to α-Gal/GalNAc from β-Gal/GalNAc terminal glycans. Based on the results, Gg preferably binds to terminal α-Gal/GalNAc than terminal β-Gal/GalNAc sugars (Fig. 3A). Comparing binding signals to glycans with similar monosaccharide components which only differ in α/β-terminal linkage showed that the binding strength is decreased to more than half with β terminal sugars (Fig. 3A). To explain this, a model of the structure of HA1 D271F mutant in complex with Gal-α-1,3-Gal and Gal-β-1,4-Glc was observed (Fig. 3B). Gg uses an HA1 W176A/D271F mutant, this serves to spontaneously knock-out the sialic acid binding site, W176A mutation, and enhance the Gal/GalNAc binding site, D271F mutation^13^. The structure of HA1 WT with β-Gal was previously reported (PDB ID, 3AH4) and β-Gal was replaced with Gal-α-1,3-Gal taken from the crystal structure of jacalin complexed with Gal-α-1,3-Gal (PDB, 5JM1) or replaced with Gal-β-1,4-Glc taken from the structure of BEL β-trefoil in complex with lactose (PDB 4I4S). Crystal structure analysis suggests that the α-Gal conformation is preferred because of the orientation of the β-linked chain which causes a direct clash with the N259 residue. The kink in the sugar chain brought about by the β- linkage hinders effective binding to the lectin. In contrast, α-linked Gal-α-1,3-Gal showed an ease of entry to the binding subsite (Fig. 3B, Supplementary Fig. S5).

**Figure 4:**
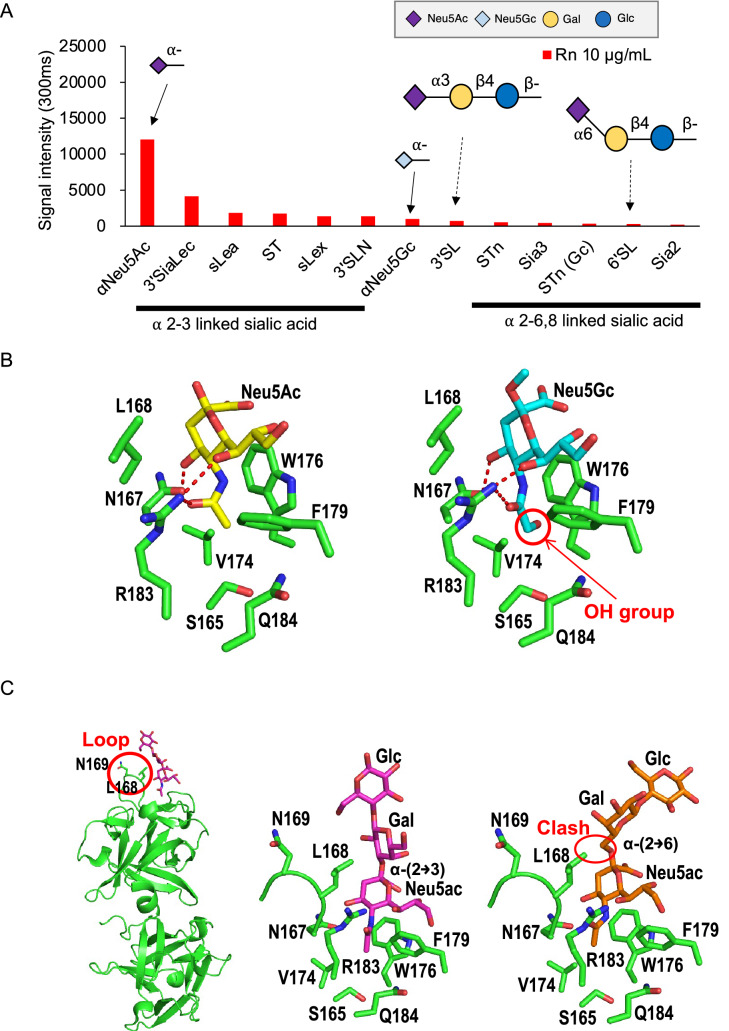
Analysis of Rn specificity. (**A**) Signal intensities of Cy3-labeled Rn to sialic acid terminal glycans. Comparison of binding to Neu5Ac and Neu5Gc are shown in solid arrows while α-(2 → 3) Neu5Ac and α-(2 → 6) Neu5Ac binding intensities are shown in dotted arrows. Cartoon representations of glycan structures are shown on top of each corresponding arrow. (**B**) Model of the area near the binding site of HA1 bound to Neu5Ac (PDB ID, 3AHI). Neu5Ac was modified to Neu5Gc and superimposed to the model. **(C)** HA1 bound to Neu5Ac model (PDB ID, 3AHI) was used and sugar was substituted with α-(2 → 3) Neu5Ac and α-(2 → 6) Neu5Ac (3SiaLac, PDB ID, 4EN6; 6SiaLac, PDB ID, 4EN8). The models of 3SiaLac and 6SiaLac were then placed in the structure of HA1 based on the position of sialic acid.

Next, we took results from Rn analysis and compared binding to sialylated glycans. Rn preferably binds to α-Neu5Ac than α-Neu5Gc residues (Fig. 4A, solid-line arrows). Looking at the models of HA1 WT in complex with Neu5Ac and Neu5Gc, the OH group of Neu5Gc makes the binding unfavorable because of steric considerations (Fig. 4B). These models were constructed by modifying the Neu5Ac ligands of a previously reported structure of HA1 complex with β-Neu5Ac (PDB ID, 3AHI). Furthermore, Rn binds to α-(2 → 3) Neu5Ac stronger compared to α-(2 → 6/8) Neu5Ac terminal ligands (Fig. 4A, dashed-line arrows). This could be explained by the structures of HA1 WT in complex with 3 sialyllactose (3’SL) and 6 sialyllactose (6’SL). Ligands (3SiaLac, PDB ID, 4EN6; 6SiaLac, PDB ID, 4EN8) were superimposed to the structure of HA1 WT based on the position of sialic acid and the HA1-Neu5Ac structure (PDB ID, 3AHI). Analysis of crystal structures show a possible clash between the α-(2 → 6) Neu5Ac from 6’SL and the L168 residue. On the other hand, α-(2 → 3) Neu5Ac in the 3’SL presents an ease in access to the subsite possibly explaining why binding affinities for sialic acid with α-(2 → 3) linkage is preferred (Fig. 4C). Ligand-bound models were further analyzed with AutoDock Vina to estimate the stability of the complexes and results support that α-linked structures bind more stably compared to β-linked Gal/GalNAc structures for Gg, while α-(2 → 3) linkage is preferred compared to α-(2 → 6) linkage for Rn (Supplementary Fig. S5).

### Confirmation of binding specificities of Gg and Rn

**Figure 5:**
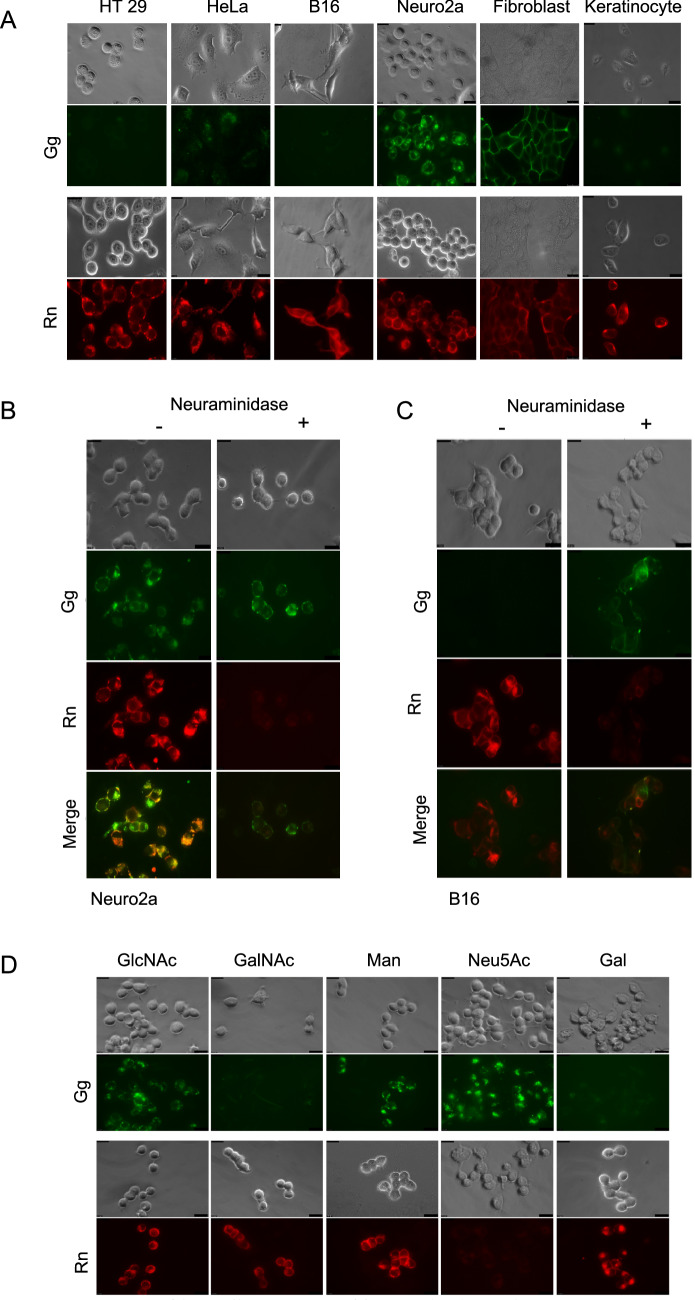
Confirmation of binding specificity of Gg and Rn on cells. **(A)** Bright field and fluorescence images of Neuro2a cells stained with Gg and Rn with and without neuraminidase. (**B**) Bright field and fluorescence images of B16F10 cells stained with Gg and Rn with and without neuraminidase. (**C**) Bright field and fluorescence images of Neuro2a cells stained with Gg and Rn in the presence of various sugars that can inhibit binding activity of lectin complex. Galactose and *N*-acetylgalactosamine inhibits binding of Gg while *N*-acetylneuraminic acid inhibits binding of Rn. Scale bars: 10 µm.

Finally, to confirm binding specificity on biological samples, Gg and Rn were stained to several cultured cells; HT29, HeLa, B16, Neuro2a, Fibroblast and keratinocytes (Fig. 5A). Different cells have different Gg and Rn binding patterns. Gg binds to HeLa, Neuro2a and fibroblast cell surfaces while Rn binds to all cell types, which is expected since most cell surfaces are known to contain sialylated glycan structures. Both Gg and Rn effectively binds to Neuro2a cells, while in B16F10 cells for example, only Rn shows distinct binding patterns. Based on these results, Neuro2a cells and B16F10 cells were used to further confirm the binding specificity of Gg and Rn in enzyme treated and sugar inhibitory analysis.

Neuro2a and B1610 cells were treated with α2-3, 6, 8-neuraminidase and then stained with Gg and Rn. Neuraminidase is an enzyme which catalyzes the hydrolysis of α2-3, α2-6, and α2-8 sialic acid residues. For Neuro2a cells, treatment with neuraminidase results to the loss of Rn staining while Gg staining remains unaffected (Fig. 5B). This confirms that Rn binds to sialic acid sugars, and removal of such in the cell surface leads to a loss in Rn staining. In addition, for B16F10 cells, although similar results were observed for Rn staining, Gg staining becomes visible after neuraminidase treatment (Fig. 5C). Neuraminidase treatment forms glycan structures with open galactose or *N*-acetylgalactosamine residues after liberation of sialic acid. The staining of Gg on B16F10 cells after treatment with neuraminidase highlights the specificity of Gg towards Gal and GalNAc sugars while the removal of Rn staining with the same treatment shows its specificity towards sialic acid residues.

In addition to neuraminidase treatment, competitive sugar inhibitory analysis was also performed (Fig. 5D). Gg and Rn were incubated with 25 mM galactose (Gal), *N*-acetylgalactosamine (GalNAc), *N*-acetylglucosamine (GlcNAc), mannose (Man), and *N*-acetylneuraminic acid (Neu5Ac) before staining to Neuro2a cells. For Gg lectin, competitive inhibition was observed for Gal and GalNAc, but not for Man, and Neu5Ac. On the other hand, Rn lectin showed competitive inhibition for Neu5Ac, but not for Gal, GalNAc, and Man. These results match well with the known specificity of HA1 W176A/D271F for Gal and GalNAc sugars, and HA1 N278A/Q279A for Neu5Ac.

Taken together these results strongly confirm the specificity of these two new lectin complexes designed using HA proteins of the *Clostridium botulinum* C16S neurotoxin complex. Rn binds to *N*-acetylneuraminic acid residues while Gg binds to galactose and *N*-acetylgalactosamine residues.

## Discussion

In this study, two new lectin complexes were produced. One that is specific to Gal/GalNAc (Gg) and another one that is specific to Sialic acid (Rn) (Fig. 1). Results of glycan microarray confirmed that Gg binds to Gal/GalNAc terminal glycans and asialylated glycoproteins (Fig. 2A). Furthermore, Gg has a higher binding avidity to ⍺-Gal/GalNAc linked structures (Fig. 3). This can be interesting because more galactosylated glycan structures contain terminal β-Gal/GalNAc compared to ⍺-Gal/GalNAc. Among the ⍺-Gal/GalNAc terminal glycans, Gg has strong binding signal to the Forssman disaccharide, Core 8, Meliobiose, Gal⍺1-3Ln and Tn. These are rare glycan structures that are not expressed by most species. Some of them, such as the Forssman disaccharide expressed in erythrocytes^21^ are also found in various cancer cells such as lung, gastric, or colon carcinoma^22,23^. The Tn epitope, as well, is also widely known for its expression in various carcinomas. In addition, the biosynthesis of several *O*-glycan core structures that are specific to Gg like Core 8 and Core 6 remains unclear^24^. Because the ligands of Gg are not commonly expressed, there is a possibility to use this lectin to study novel functions or biosynthetic pathways of glycans.

In contrast, Rn was found to be specific to sialylated glycans and glycoproteins with a preference to *N*-acetylneuraminic acid (Neu5Ac) and ⍺ (2-3) linked sialic acid residues (Figs. 2B, 4). The sugar binding specificities were further explained from results of HA1-ligand bound models (Fig. 4). Rn’s specificity exclusively to Neu5Ac residues suggests that this lectin complex can be used to compare sialylated glycans in human tissue compared to other species, since humans lack *N*-glycol neuraminic acid (Neu5Gc). Moreover, Rn has a strong binding signal to terminal ⍺(2-3) linked sialic acid residues. Among these structures, glycans SLe^x^, Sle^a^, and ST are structures that are overexpressed in cancer^25^. The construction and confirmation of a lectin that discriminates between ⍺(2-3) and ⍺(2-6) linked sialic acid is important especially because these sialylated glycans are differentially expressed in cancer^26^. Furthermore, normal cells have modifications in long-branched sugar chains, whereas cancer cells express various simple and short sugar chain antigens called *O*-glycans (e.g., Tn, sialyl-Tn and sialyl-Lewis-X), these antigens were reported to have strong binding signals with Gg and Rn, making it an interesting avenue for future applications of Gg and Rn to study cancer cells.

Of interesting note as well is the possible novelty of these lectins in terms of binding specificity to various cells and tissues. There are many galactose and sialic acid specific lectins, however few sialic acid specific lectins can distinguish between ⍺(2-3) and ⍺(2-6). Similarly, only few galactose-specific lectins are specific to ⍺-Gal/GalNAc. Because of the binding strength of the complex, as shown by clear cell staining results with a short reaction time, as well as its preference to rare glycan structures, it will be interesting to compare binding patterns of Gg and Rn with other known lectins that are specific to Gal/GalNAc or Sialic acid. Difference in binding patterns can possibly provide a better understanding on the formation or heterogeneity of glycan structures.

The binding specificities of Gg and Rn were confirmed in Fig. 5. The membranes of cells are well decorated with glycoconjugates that end in different types of sugars. Glycosylation patterns are known to change between species, with development and in the onset or progression of various diseases. Our results showed that Gg and Rn have different binding patterns depending on the cell line used. The more common glycoconjugates that decorate cellular membranes are the complex type glycoproteins that end in Neu5Ac terminal residues, this explains why Rn stains majority of the different types of cells (Fig. 5A). Neuro2a cells are both stained with Gg and Rn, although different binding patterns were observed. This is interesting because Neuro2a cells have various applications in neurobiology, neurotoxicity and even in Alzheimer’s disease studies. The staining of our lectins to Neuro2a opens up the potential of using our lectin for brain imaging and other neuro-glycobiology studies. In contrast, B16F10 cells is only stained by Rn. These two cell lines were then used to confirm the specificity of Gg and Rn. When Neuro2a and B16F10 cells were treated with neuraminidase, the staining of Rn was lost which confirms specificity to Neu5Ac (Fig. 5B,C). It is interesting to note as well that even on cells which are stained by both Gg and Rn, microdomains are formed which further confirm that Gg and Rn have different specificities and bind to different ligands within the membrane (Fig. 5B,C, merge). Furthermore, the release of Neu5Ac sugars after neuraminidase treatment of B16F10 cells resulted to the exposure of Gal terminal sugars which were stained by Gg (Fig. 5C). To further evaluate the carbohydrate-binding specificity of Gg and Rn, inhibition experiments were performed. For Gg, incubation with Gal and GalNAc showed inhibition of cell staining by binding competitively to the lectin. For Rn, inhibition was observed by competitive binding of Neu5Ac sugar to the lectin (Fig. 5D).

The interaction between proteins and sugars are usually weak. However, when 2 or more sugar chains bind to a protein the interaction is enhanced. The enhanced binding which results from the accumulation of 10 or more sugar chains, which are multivalent compounds, is known as the glycocluster effect^27^. These multivalent compounds exist in cell surfaces such as raft like structures of glycolipids and from glycoproteins. Glycoproteins have a dendritic structure of sugar chains and thus multivalent effects are more likely to occur. Furthermore, proteins can have multiple sugar binding sites that allow simultaneous binding of multivalent ligands to multiple binding sites. Examples of these are concavalin A which has four binding sites and the Shiga toxin with fifteen binding sites. These factors contribute to an enhanced protein–sugar interaction^27^. Our lectins Gg and Rn have 12 possible binding sites each that can bind to naturally occurring multivalent sugar chains from glycoproteins localized in the cell membrane. Furthermore, the botulinum neurotoxin complex is a potent neurotoxin that can express its toxicity in the body because of the stability given by its association with nontoxic proteins (HA1, HA2, and HA3)^28^. The HA proteins, therefore, form a stable complex that retains its structure even in harsh conditions such as that of the gastrointestinal tract. With these factors in consideration, the increased number of binding sites, the structure of the complex, and the directed mutagenesis possibly explains the strong and highly specific binding of the lectin complexes produced.

The new lectin complexes Gg and Rn provide us with an easy to use, one-step chemical that can be used as a tool to study glycans in cells and different tissues. These complexes are stable, specific, and provide clear cell staining results with a short reaction time. Because of the above-mentioned properties, Gg and Rn can be powerful tools to study glycan structure and formation. It will be interesting to investigate the application of these lectin complexes in screening different disease-specific cells such as cancer or in glycan formation studies of complex tissues such as those involved in the nervous system. This is currently the on-going theme of our research.

## Materials and methods

### Materials

QuikChange directed mutagenesis kit was purchased from Stratagene (Garden Grove, CA, USA). Amylose resin and enterokinase light chain (P8070S) were purchased from New England Biolabs Inc. (Ipswich, MA, USA). Nickel-nitrilotriacetic acid (Ni–NTA) agarose were purchased from QIAGEN (No. 30210). Amicon Ultra 10 kDa (No. ACS501024) Merck Millipore (Darmstadt, Germany). Fluorescein labels Alexa Flour 488 (A20000) and Alexa Flour 594 (A20004) were purchased from Thermo Fisher Scientific (Waltham, MA, USA). PD10 column (17-0851-01) used for buffer exchange were purchased from GE healthcare (Chicago, IL, USA). Dulbecco's modified Eagle's medium, *N*-Acetyl-_D_-galactosamine (A2795) and _D_-galactose (G0750) were purchased from Sigma (St. Louis, MO, USA). RPMI-1640 was purchased from Invitrogen/ThermoFisher Scientific (Waltham, MA, USA). Mannose was purchased from Hayashibara Co. Ltd., (Okayama, Japan). Chamber slides for cell culture (8-well chamber) were purchased from IWAKI (Chiba, Japan) and *N*-acetylneuraminic acid (08371-36), *N*-Acetyl-_D_-glucosamine (00520) and Luria broth, miller (20068) were obtained from Nacalai Tesque (Kyoto, Japan).

### Construction of HA1-2-3 complex

The expression and purification of HAs have been previously reported^17,18^. In brief, HA1 was expressed as a maltose binding protein (MBP)-2xFLAG-HA1 fusion protein in *Escherichia coli* (*E.coli*) JM109 cells cultured in Luria broth (SIGMA) and purified by affinity chromatography using amylose resin (New England Biolabs Inc).

After cleavage of the fusion protein by enterokinase (New England Biolabs Inc.), the sample was applied to Benzamidine-Sepharose® 6B (GE Healthcare) to separate the cleaved products from enterokinase. The cleaved products were then purified by affinity chromatography using amylose resin to separate the MBP-2 × FLAG tag from HA1 protein.

The purified HA1 was incubated with HA2 at 27℃ for 18 h. HA2 was expressed as 6xHis-T7-2xFLAG fusion protein(6xHis-T7-2xFLAG-HA2)in *E.coli* BL21(DE3) cells. HA1-HA2 complex was purified by affinity chromatography using Ni-NTAagarose (QIAGEN) and eluted using 100 to 200 mM imidazole (wash buffer: 20 mM Tris–HCl, 50 mM NaCl, pH 8.0; elution buffer: 20–200 mM imidazole diluted in wash buffer). The purified HA1-2 complex was applied to a 12% SDS polyacrylamide gel and electrophoresis was performed at room temperature for approximately 45 min using constant current (15 mA) and detected afterwards by Coomassie Brilliant Blue (CBB) staining. The binding ratio was checked by calculating the band intensity detected by lumino image analyzer LAS-3000 (FUJIFILM) and Multi gauge ver. 2.1 (FUJIFILM).

HA3 was expressed and purified in a similar way, although fusion protein was kept intact. MBP-2 × FLAG-HA3 fusion protein was incubated with HA1-HA2 complex at 27℃ for 18 h and purified by Ni–NTA chromatography, followed by amylose resin chromatography using the same methods as mentioned above. Finally, HA1-HA2-HA3 complex (HA complex) was centrifuged at 4000 rpm using Amicon Ultra 10 KDa (Merck Millipore) to concentrate the solution, and the concentration of the complex checked by analyzing SDS-PAGE gels with LAS-3000 and Multi gauge ver. 2.1 (FUJIFILM). A schematic diagram of the construction of the HA1-2–3 complex is shown in Supplementary Fig. S1. Two lectin complexes were made and are named as follows; Gg: HA complex which has HA1 W176A/D271F and labeled with Alexa Flour 488 and Rn: HA complex which has HA1 N278A/Q279A and labeled with Alexa Flour 594.

### Glycoconjugate microarray production and analysis

Glycoconjugate microarrays were produced as previously described^29^. Glycoproteins and glycoside-polyacrylamide (PAA) conjugates were dissolved in a spotting solution (Matsunami Glass Ind., LTD., Osaka, Japan) at a final concentration of 0.5 and 0.1 mg/mL, respectively (Supplementary Fig. S4). After filtration using 0.22 µm poresize filter to remove insoluble particles, they were spotted on a microarray-grade epoxy-coated glass slide (Schott AG, Mainz, Germany) attached with a silicone rubber sheet with 7 chambers, using a non-contact microarray printing robot (MicroSys 4000; Genomic solutions Inc, MI, USA). Glycoproteins were immobilized on epoxy-coated glass slides by amine coupling via the amino groups present in glycoproteins. Previously, glycoside-PAA conjugates were demonstrated to be efficiently immobilized on epoxy-coated glass slides, but its mechanism has not been clarified yet^29^. The glass slide was incubated in a humidity-controlled incubator at 25 °C for 3 h to allow immobilization. After incubation, excess amounts of non-immobilized materials were washed out with probing buffer (25 mM Tris–HCl, pH 7.4 containing 0.8% NaCl, 1% (v/v) Triton-X, 1 mM MnCl_2_, 1 mM CaCl_2_), and blocked with 100 µL of TBS (25 mM Tris–HCl, pH 7.4 containing 0.8% NaCl) containing 1% BSA at 20 °C for 1 h. Lectin complexes (10 µg/mL) were labeled with Cy3 in probing buffer and incubated with the glass slides (80 µL/well) at 20℃ overnight. After which, fluorescent images were immediately acquired using a Bio-Rex scan 200 evanescent-field activated fluorescence scanner (Rexxam Co. Ltd., Kagawa, Japan). The net intensity value for each spot was determined by signal intensity minus the background value. Data are the average ± S.D. of triplicate spots.

### Ligand-bound models of HA1

We constructed ligand-bound models of HA1 in complex with α-Neu5Ac and α-Neu5Gc using previously reported structure of HA1 complex with β-Neu5Ac (PDB ID, 3AHI). The coordinate of α-Neu5Ac was obtained from the CCP4 monomer library and the coordinate of α-Neu5Gc was taken from PDB 3TAY, and then the ligands were replaced based on the position of pyranose ring with the program Coot. The HA1 model in complex with 3’SiaLac and 6’SiaLac was constructed using HA1 PDB 3AHI structure, and 3’SiaLac and 6’SiaLac were obtained from previously reported HA70-sialyllactose complexes (HA70/C-3’SiaLac, PDB ID, 4EN6; HA70/C-6’SiaLac, PDB ID, 4EN8). The models of 3’SiaLac and 6’SiaLac were then placed in the structure of HA1 based on the position of Neu5Ac. The HA1 WADF mutant with Gal-α-1,3-Gal and lactose were constructed using the structure of HA1 WT complexed with Gal (PDB ID, 3AH4) superimposed with ligands from previously published reports, Gal-α-1,3-Gal (PDB 5JM1) and lactose (PDB 414S). Mutation changing residue D271 to Phe was prepared by choosing the most sterically favored rotamer in Coot program and the ligands were substituted based on the position of Gal. The amino acid residues involved in the binding to the ligands were visualized using PyMOL software. Stability of the complexes were estimated using the program Autodock Vina.

### Cytochemical staining with lectins

Neuro2a, B16F10, HT29, human epidermal keratinocyte cell line (HaCaT), human fibroblast (fibroblast), and human keratinocyte (keratinocyte) cells were incubated in Dulbecco’s modified Eagle’s medium (Sigma, St. Louis, MO, USA) containing 10% fetal bovine serum (FBS) and 0.1 mg/mL kanamycin at 37 °C in a humidified incubator supplied with 5% CO_2_ in air. Human cervical cancer-derived cell line HeLa cells were cultured in Roswell Park Memorial Institute (RPMI) 1640 (ThermoFisher Scientific Waltham, MA) containing 10% fetal bovine serum (FBS) at 37 °C in a humidified incubator supplied with 5% CO_2_ in air. The cell solution (100 μL) was plated at an initial density of 1.0 × 10^4^ cells/ml on coverslips (ThermoFisher Scientific Waltham, MA), and placed in 8-well cell-culture plates coated with collagen (IWAKI). The cells were incubated for 24 h before examination and were washed three times with PBS buffer at 0.5 mL/well (washing step). Unbound sites were blocked by incubation with 100 μL of 1.0% (w/v) bovine serum albumin in PBS for 30 min. After the blocking solution was removed, the wells were incubated with 100 µL of Gg (2 µg/ mL) or Rn (2 µg/mL) in blocking solution for 1 min at room temperature. The lectin solution was then removed by aspiration and cells were washed with PBS three times. Fresh culture media (100 µL) was added to each well and fluorescent images were taken and analyzed with Leica DMI AF6000 B (Leica microsystems).

### Neuraminidase treatment

Neuro2a and B16F10 cells were used, and 0.5 units/mL neuraminidase (New England Biolabs) was added, and the mixture was incubated for 1 h at 37 ℃. After washing with PBS, cells were incubated with Gg and Rn following the above-mentioned procedure. The incubated cells were washed with PBS and fluorescent images were analyzed with Leica DMI AF6000 B (Leica microsystems).

### Competitive sugar inhibition assay

To study the specificity of lectin by competition or inhibition of lectin binding to Neuro2a cells, 2 μg/mL of each individual lectin complex was mixed with 25 mM  competitive sugar. Neuro2a cells were washed with PBS three times, then incubated with sugar-treated lectins for 3 min at 37 °C. The following monosaccharide were used as inhibitors: *N*-Acetyl-_D_-galactosamine (SIGMA), *N*-Acetyl-_D_-glucosamine (Nacalai Tesque), Mannose (HAYASHIBARA Co. Ltd.), *N*-acetylneuraminic acid (Nacalai Tesque) or Galactose (SIGMA). After incubation, Gg or Rn was aspirated out, cells were washed with PBS and 100 µL culture medium was added. Staining patterns were observed using fluorescence microscopy as described above.

## Supplementary Information

Supplementary Figures.
