# Supplementary material for: Glycan detecting tools developed from the Clostridium botulinum whole hemagglutinin complex
Source: Sci Rep. 2021 Nov 9;11:21973. doi: 10.1038/s41598-021-01501-1 (PMC8578614; doi:10.1038/s41598-021-01501-1)
Supplement: Supplementary file 1 — Supplementary Figures. [file 41598_2021_1501_MOESM1_ESM.pdf]

Supplementary Information for:

Glycan Detecting Tools Developed from the *Clostridium botulinum* Whole Hemagglutinin Complex

Ea Kristine Clarisse Tulin[1], Chiaki Nakazawa[2], Tomomi Nakamura[2], Shion Saito[2], Naoki Ohzono[2], Shin-ichi Nakakita[3], Keiko Hiemori[4], Hiroaki Tateno[4], Takashi Tonozuka[2], and Atsushi Nishikawa[1,2]*

[1]United Graduate School of Agricultural Science, Tokyo University of Agriculture and Technology, Tokyo, 183-8509, Japan.
[2]Department of Applied Biological Chemistry, Graduate School of Agriculture, Tokyo University of Agriculture and Technology, Tokyo, 183-8509, Japan.
[3]Division of Functional Glycomics, Kagawa University, Kagawa, 760-0016, Japan.
[4]Cellular and Molecular Biotechnology Research Institute, National Institute of Advanced Industrial Science and Technology, Tsukuba, 305-8566, Japan.

*corresponding: nishikaw@cc.tuat.ac.jp

HA1-2-3 Complex (Gg/Rn)

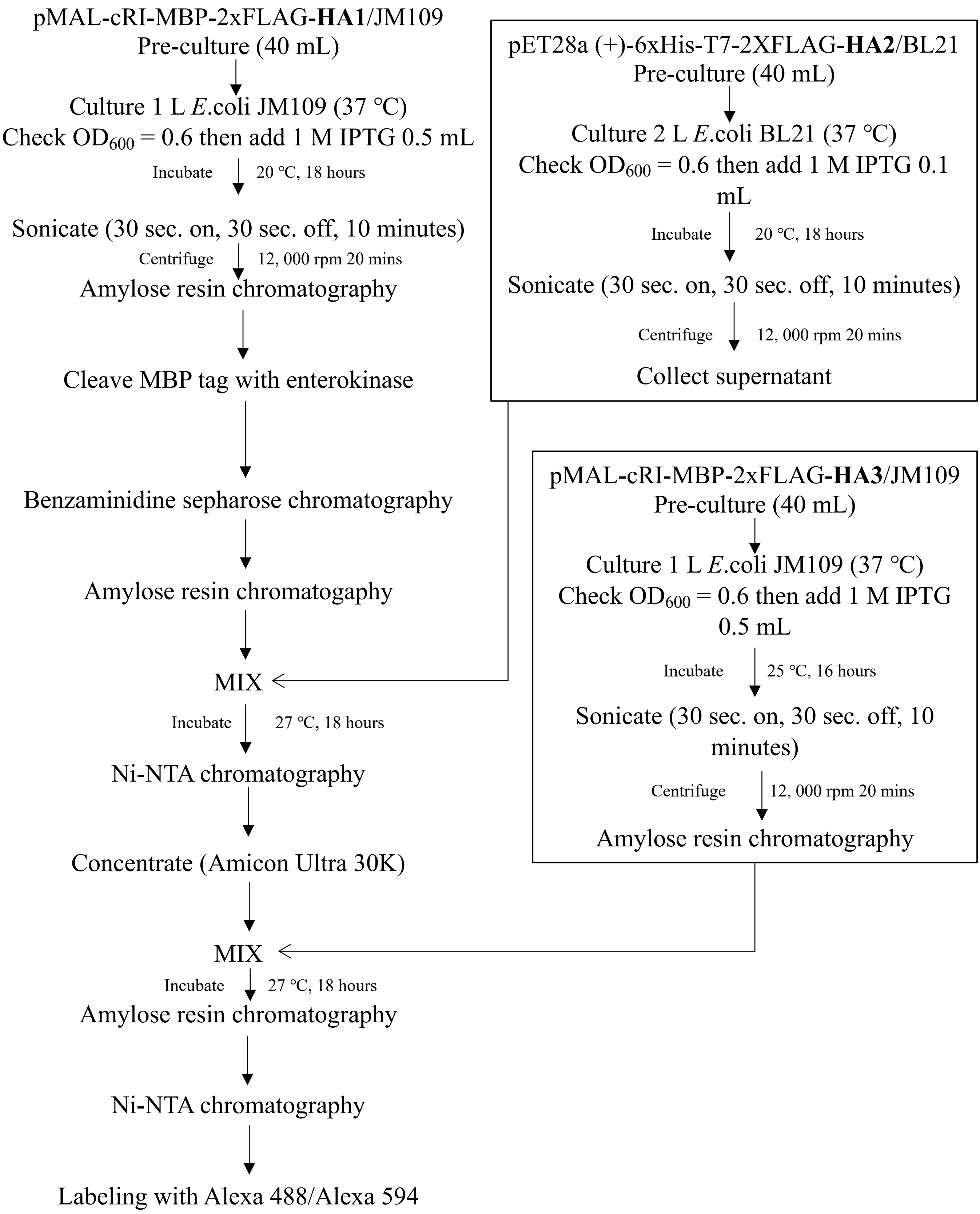

Supplementary Figure S1. Schematic diagram of Gg and Rn preparation.

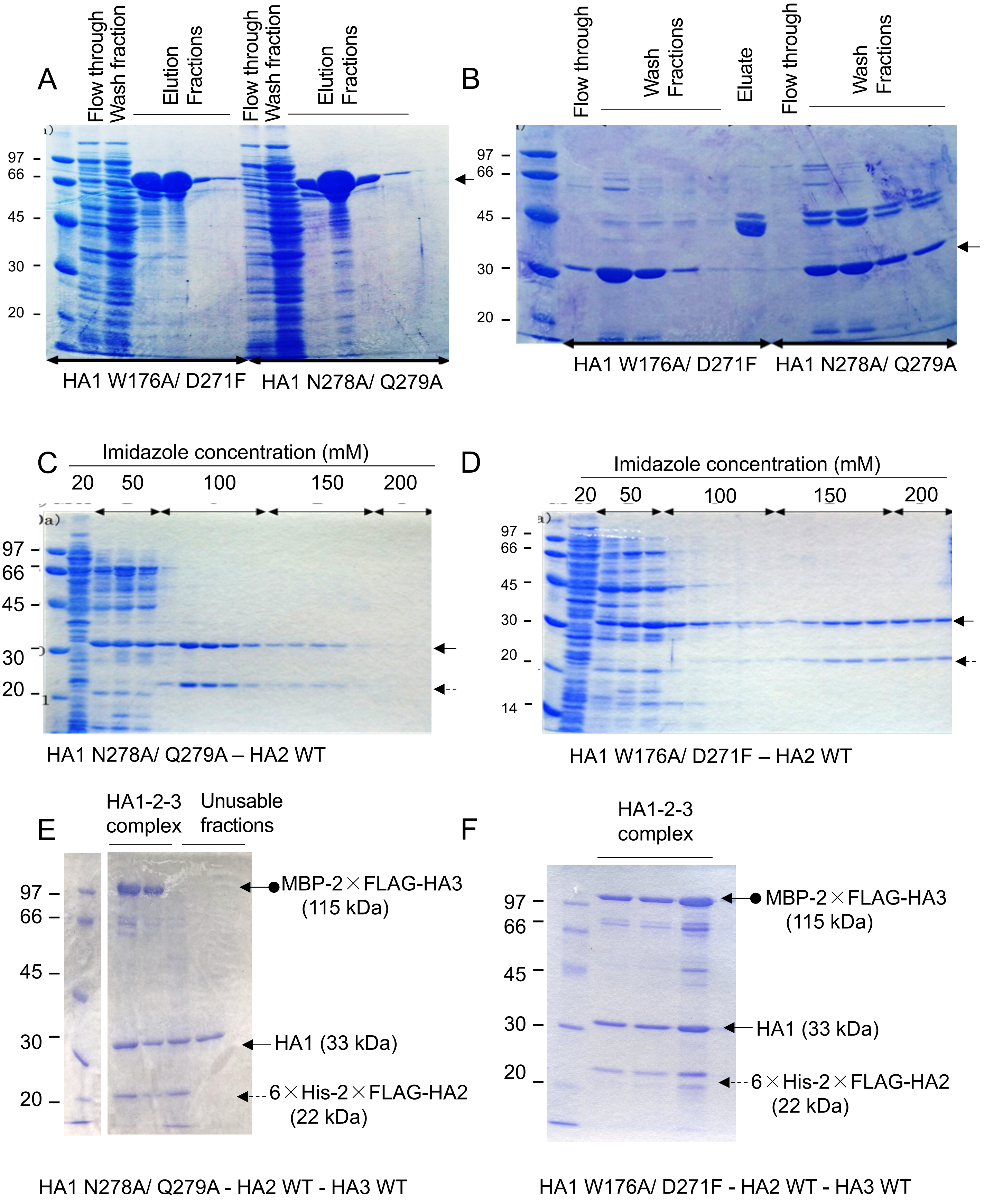

Supplementary Figure S2. Preparation of HA complex. The full-length gels of Fig. 1A. Purification of (A) MBP-HA1, (B) HA1 only, (C-D) HA1-2 complex purification, and (E-F) HA1-2-3 complex for both mutants are shown. Target proteins are labeled as HA1 (←), HA2 (←--), and MBP-HA3 (←●).

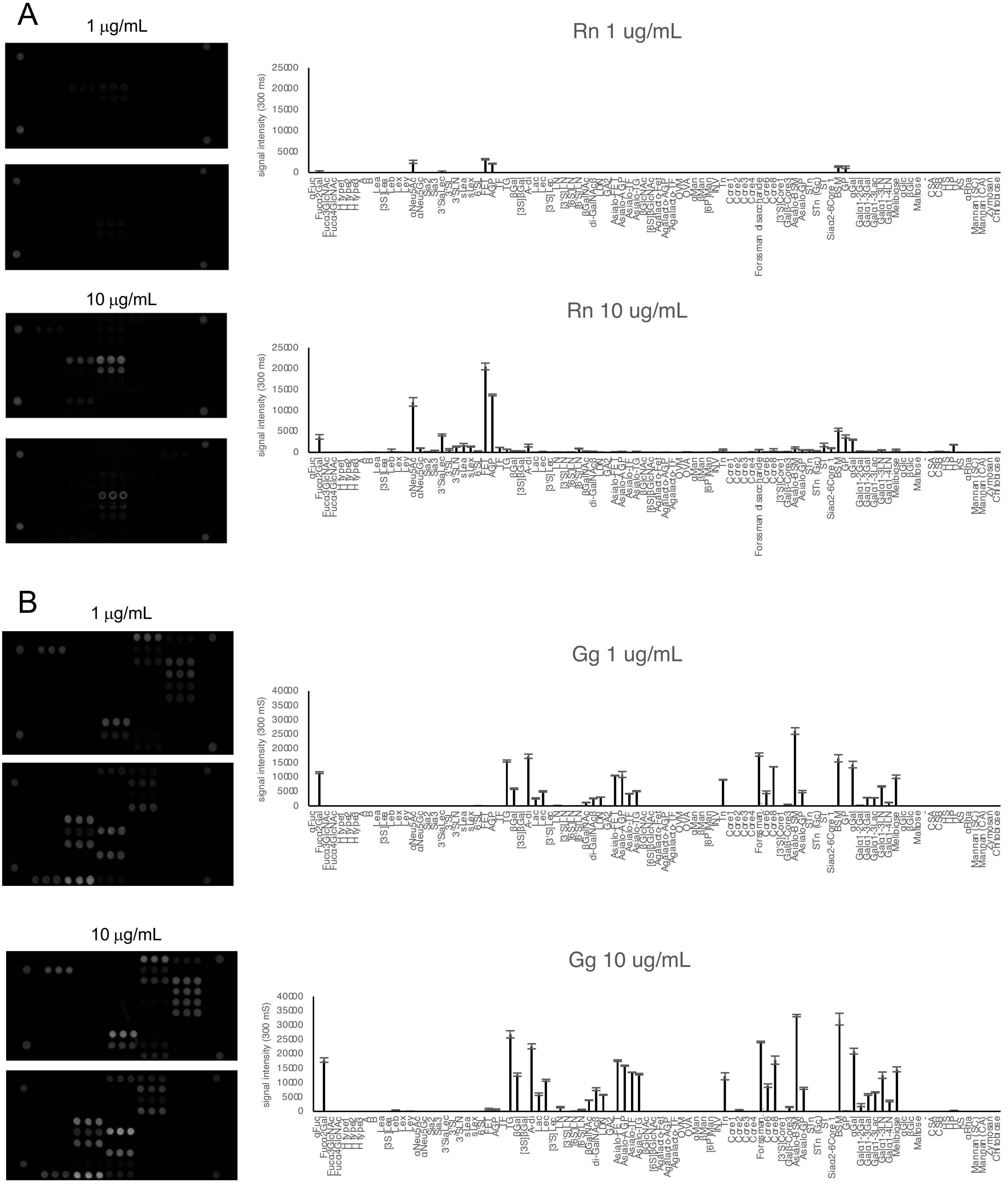

Supplementary Figure S3. Binding of (A) Gg and (B) Rn on the glycan array at two different concentrations, with the actual image of the glycan microarray plate shown and the plot of binding.

Number	Trivial name	Presentation	Glycans
1	αFuc	PAA	Fucα1-PAA
2	Fucα2Gal	PAA	Fucα1-2Galβ1-PAA
3	Fucα3GlcNAc	PAA	Fucα1-3GlcNAcβ1-PAA
4	Fucα4GlcNAc	PAA	Fucα1-4GlcNAcβ1-PAA
5	H type1	PAA	Fucα1-2Galβ1-3GlcNAcβ1-PAA
6	H type2	PAA	Fucα1-2Galβ1-4GlcNAcβ1-PAA
7	H type3	PAA	Fucα1-2Galβ1-3GalNAcα1-PAA
8	A	PAA	GalNAcα1-3(Fucα1-2)Galβ1-4GlcNAcβ1-PAA
9	B	PAA	Galα1-3(Fucα1-2)Galβ1-4GlcNAcβ1-PAA
10	Le^{a}	PAA	Galβ1-3(Fucα1-4)GlcNAcβ1-PAA
11	[3S]Le^{a}	PAA	(3OSO$_3$)Galβ1-3(Fucα1-4)GlcNAcβ1-PAA
12	Le^{b}	PAA	Fucα1-2Galβ1-3(Fucα1-4)GlcNAcβ1-PAA
13	Le^{x}	PAA	Galβ1-4(Fucα1-3)GlcNAcβ1-PAA
14	Le^{y}	PAA	Fucα1-2Galβ1-4(Fucα1-3)GlcNAcβ1-PAA
15	αNeu5Ac	PAA	Neu5Acα2-PAA
16	αNeu5Gc	PAA	Neu5Gcα2-PAA
17	Sia2	PAA	Neu5Acα2-8Neu5Acα2-PAA
18	Sia3	PAA	Neu5Acα2-8Neu5Acα2-8Neu5Acα2-PAA
19	3'SiaLe^{c}	PAA	Neu5Acα2-3Galβ1-3GlcNAcβ1-PAA
20	3'SL	PAA	Neu5Acα2-3Galβ1-4Glcβ1-PAA
21	3'SLN	PAA	Neu5Acα2-3Galβ1-4GlcNAcβ1-PAA
22	sLe^{a}	PAA	Neu5Acα2-3Galβ1-3(Fucα1-4)GlcNAcβ1-PAA
23	sLe^{x}	PAA	Neu5Acα2-3Galβ1-4(Fucα1-3)GlcNAcβ1-PAA
24	6'SL	PAA	Neu5Acα2-6Galβ1-4Glcβ1-PAA
25	FET	Glycoprotein	Fetuin (complex-type N-glycans and O-glycans)
26	AGP	Glycoprotein	α1-acid glycoprotein (complex-type N-glycans)
27	TF	Glycoprotein	Transferrin (complex-type N-glycans)
28	TG	Glycoprotein	Porcine thyroglobulin (complex and high-mannnose-type N-glycans, and O-glycans)
29	βGal	PAA	Galβ1-PAA
30	[3S]βGal	PAA	(3OSO$_3$)Galβ1-PAA
31	A-di	PAA	GalNAcα1-3Galβ1-PAA
32	Lac	PAA	Galβ1-4Glcβ1-PAA
33	Le^{c}	PAA	Galβ1-3GlcNAcβ1-PAA
34	[3'S]Le^{c}	PAA	(3OSO$_3$)Galβ1-3GlcNAcβ1-PAA
35	LN	PAA	Galβ1-4GlcNAcβ1-PAA
36	[3'S]LN	PAA	(3OSO$_3$)Galβ1-4GlcNAcβ1-PAA
37	[6S]LN	PAA	Galβ1-4(6OSO$_3$)GlcNAcβ1-PAA
38	[6'S]LN	PAA	(6OSO$_3$)Galβ1-4GlcNAcβ1-PAA
39	βGalNAc	PAA	GalNAcβ1-PAA
40	di-GalNAcβ	PAA	GalNAcβ1-3GalNAcβ1-PAA
41	LDN	PAA	GalNAcβ1-4GlcNAcβ1-PAA
42	GA2	PAA	GalNAcβ1-4Galβ1-4Glcβ1-PAA
43	Asialo-FET	Glycoprotein	Asialo fetuin (desialylated complex-type N- and O-glycans)
44	Asialo-AGP	Glycoprotein	Asialo α1-acid glycoprotein (desialylated complex-type N-glycans)
45	Asialo-TF	Glycoprotein	Asialo transferrin (desialylated complex-type N-glycans)
46	Asialo-TG	Glycoprotein	Asialo porcine thyroglobulin (desialylated complex-type N-glycans, high-mannose-type N-glycans)
47	βGlcNAc	PAA	GlcNAcβ1-PAA
48	[6S]βGlcNAc	PAA	(6OSO$_3$)GlcNAcβ1-PAA
49	Agalacto-Fet	Glycoprotein	Agalacto fetuin (agalactosylated complex-type N- and O-glycans)
50	Agalacto-AGP	Glycoprotein	Agalacto α1-acid glycoprotein (agalactosylated complex-type N- and O-glycans)
51	Agalacto-TF	Glycoprotein	Agalacto transferrin (agalactosylated complex-type N-glycans, high-mannose-type N-glycans)
52	OVM	Glycoprotein	Ovomucoid (complex-type N-glycans)
53	OVA	Glycoprotein	Ovoalbumin (hybrid-type N-glycans)
54	αMan	PAA	Manα1-PAA
55	βMan	PAA	Manβ1-PAA
56	[6P]Man	PAA	(6OPO$_4$)Manα1-PAA
57	INV	Glycoprotein	Yeast invertase (high mannose-type N-glycans)
58	Tn	PAA	GalNAcα1-PAA
59	Core1	PAA	Galβ1-3GalNAcα1-PAA
60	Core2	PAA	Galβ1-3(GlcNAcβ1-6)GalNAcα1-PAA
61	Core3	PAA	GlcNAcβ1-3GalNAcα1-PAA
62	Core4	PAA	GlcNAcβ1-3(GlcNAcβ1-6)GalNAcα1-PAA
63	Forssman disaccharide	PAA	GalNAcα1-3GalNAcβ1-PAA
64	Core6	PAA	GlcNAcβ1-6GalNAcα1-PAA
65	Core8	PAA	Galα1-3GalNAcα1-PAA
66	[3'S]Core1	PAA	(3OSO$_3$)Galβ1-3GalNAcα1-PAA
67	Galβ-Core3	PAA	Galβ1-4GlcNAcβ1-3GalNAcα1-PAA
68	Asialo-BSM	Glycoprotein	Asialo bovine submaxillary mucin (Tn)
69	Asialo-GP	Glycoprotein	Asialo human glycophorin MN (T)
70	STn	PAA	Neu5Acα2-6GalNAcα1-PAA
71	STn (Gc)	PAA	Neu5Gcα2-6GalNAcα1-PAA
72	ST	PAA	Neu5Acα2-3Galβ1-3GalNAcα1-PAA
73	Siaα2-6Core1	PAA	Galβ1-3(Neu5Acα2-6)GalNAcα1-PAA
74	BSM	Glycoprotein	Bovine submaxillary mucin (Sialyl Tn)
75	GP	Glycoprotein	Human glycophorin (Disialyl T and sialyl Tn)
76	αGal	PAA	Galα1-PAA
77	Galα1-2Gal	PAA	Galα1-2Galβ1-PAA
78	Galα1-3Gal	PAA	Galα1-3Galβ1-PAA
79	Galα1-3Lac	PAA	Galα1-3Galβ1-4Glcβ1-PAA
80	Galα1-3LN	PAA	Galα1-3Galβ1-4GlcNAcβ1-PAA
81	Galα1-4LN	PAA	Galα1-4Galβ1-4GlcNAcβ1-PAA
82	Melibiose	PAA	Galα1-6Glcβ1-PAA
83	αGlc	PAA	Glcα1-PAA
84	βGlc	PAA	Glcβ1-PAA
85	Maltose	PAA	Glcα1-4Glcβ1-PAA
86	-	-	-
87	CSA	BSA	Chondoroitin sulfate A-BSA
88	CSB	BSA	Chondoroitin sulfate B-BSA
89	HS	BSA	Heparan sulfate-BSA
90	HP	BSA	Heparin-BSA
91	KS	BSA	Keratan sulfate-BSA
92	αRha	PAA	Rhamnoseα1-PAA
93	Mannan (SC)	Glycoprotein	*S. cerevisiae* mannan
94	Mannan (CA)	Glycoprotein	*C.albicans* mannan
95	Zymosan	Glycoprotein	Zymosan
96	Chitobiose	PAA	GlcNAcβ1-4GlcNAcβ1-PAA
97	BSA	BSA	-
98	Negative PAA	PAA	-
99	Marker		
100	BG		

Supplementary Figure S4. List of all the glycan structures in the microarray

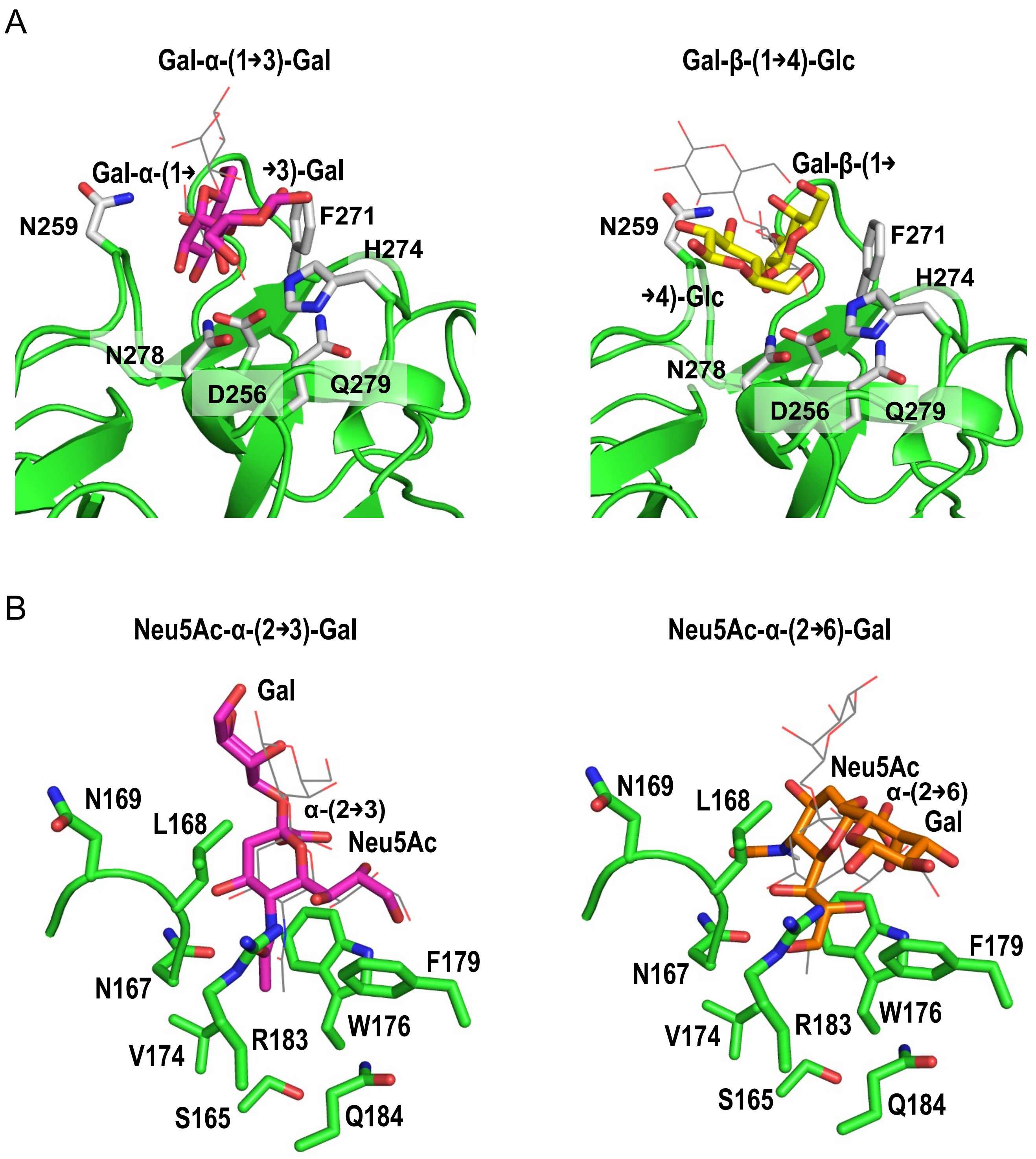

Supplementary Figure S5. Ligand-bound model simulation with AutoDock Vina. **(A)** HA1-D270F model bound to Gal-α-1,3-Gal and Gal-β-1,4-Glc ligands. For Gal-α-1,3-Gal, the galactose residue at the reducing end was predicted to interact with His274 and directed towards the outside of the HA1 molecule, indicating that longer glycans having an α-galactose residue at the non-reducing end appear to bind stably to D271F. On the other hand, glucose residue at the reducing end of lactose was predicted to be present at the molecular surface of HA1, suggesting that binding of longer glycans having a β-galactose residue at the non-reducing end is less stable due to the steric hindrance. **(B)** Neu5Ac residue in Neu5Ac-α-2,3-Gal was located in the sugar binding pocket, suggesting that Neu5Ac-α-2,3-Gal binds stably to HA1. In contrast, Neu5Ac residue of Neu5Ac-α-2,6-Gal was not present in the sugar binding pocket, suggesting that binding with Neu5Ac-α-2,6- structure is not accommodated.
